# Natural killer T (NKT) cells accelerate Shiga toxin type 2 (Stx2) pathology in mice

**DOI:** 10.3389/fmicb.2015.00262

**Published:** 2015-04-08

**Authors:** Fumiko Obata, Priyanka B. Subrahmanyam, Aimee E. Vozenilek, Lauren M. Hippler, Tynae Jeffers, Methinee Tongsuk, Irina Tiper, Progyaparamita Saha, Dakshina M. Jandhyala, Glynis L. Kolling, Olga Latinovic, Tonya J. Webb

**Affiliations:** ^1^Department of Microbiology and Immunology, University of Maryland School of MedicineBaltimore, MD, USA; ^2^Department of Molecular Pathology, University of Yamanashi Graduate School of MedicineChuo, Japan; ^3^Department of Molecular Biology and Microbiology, Tufts UniversityBoston, MA, USA; ^4^Division of Infectious Diseases and International Health, Department of Medicine, University of VirginiaCharlottesville, VA, USA; ^5^Institute of Human Virology, University of Maryland School of MedicineBaltimore, MD, USA

**Keywords:** Shiga toxin, *Escherichia coli*, hemolytic uremic syndrome, natural killer T cell, cytokines, single molecule imaging, STORM-TIRF, mouse models

## Abstract

Shiga toxin-producing *Escherichia coli* (STEC) is a leading cause of childhood renal disease Hemolytic Uremic Syndrome (HUS). The involvement of renal cytokines and chemokines is suspected to play a critical role in disease progression. In current article, we tested the hypothesis that NKT cells are involved in Stx2-induced pathology *in vivo*. To address this hypothesis we compared Stx2 toxicity in WT and CD1 knockout (KO) mice. In CD1KO mice, which lack natural killer T (NKT) cells, Stx2-induced pathologies such as weight loss, renal failure, and death were delayed. In WT mice, Stx2-specific selective increase in urinary albumin occurs in later time points, and this was also delayed in NKT cell deficient mice. NKT cell-associated cytokines such as IL-2, IL-4, IFN-γ, and IL-17 were detected in kidney lysates of Stx2-injected WT mice with the peak around 36 h after Stx2 injection. In CD1KO, there was a delay in the kinetics, and increases in these cytokines were observed 60 h post Stx2 injection. These data suggest that NKT cells accelerate Stx2-induced pathology in mouse kidneys. To determine the mechanism by which NKT cells promote Stx2-associated disease, *in vitro* studies were performed using murine renal cells. We found that murine glomerular endothelial cells and podocytes express functional CD1d molecules and can present exogenous antigen to NKT cells. Moreover, we observed the direct interaction between Stx2 and the receptor Gb_3_ on the surface of mouse renal cells by 3D STORM-TIRF which provides single molecule imaging. Collectively, these data suggest that Stx2 binds to Gb_3_ on renal cells and leads to aberrant CD1d-mediated NKT cell activation. Therefore, strategies targeting NKT cells could have a significant impact on Stx2-associated renal pathology in STEC disease.

## Introduction

Shiga toxin-producing *Escherichia coli* (STEC) causes renal disease, hemolytic uremic syndrome (HUS) (Karmali et al., 1985). HUS is a leading cause of renal failure in otherwise healthy children and Shiga toxin type 2 (Stx2) is often associated with severe symptoms of STEC infection (Lopez et al., 1989; Ostroff et al., 1989). In STEC patients, an increase in white blood cells (WBC) namely neutrophils and monocytes, is often associated with HUS (Gianantonio et al., 1973; Ryan et al., 1986; Martin et al., 1990; Robson et al., 1993; Su and Brandt, 1995; Perez et al., 1998; Dervenoulas et al., 2000; Ake et al., 2005; Fernandez et al., 2005; Ramos et al., 2007). Serum cytokines such as IL-6, IL-8, IL-10 and TNFα are often found increased in STEC-HUS (Murata et al., 1998; Proulx et al., 1998; Shimizu et al., 2012; Valles et al., 2012). Similarly, in animal models, Shiga toxins-injected animals showed blood profiles such as an increase in neutrophils (Fernandez et al., 2006; Keepers et al., 2006; Sauter et al., 2008), IL-6 and TNFα (Sauter et al., 2008; Stearns-Kurosawa et al., 2010). In addition, several animal model studies have found a variety of cytokines and chemokines are produced in the kidney (Keepers et al., 2007; Roche et al., 2007; Sauter et al., 2008; Zanchi et al., 2008; Petruzziello-Pellegrini et al., 2012; Stearns-Kurosawa et al., 2013). The mechanism by which Shiga toxins rapidly induce many types of cytokines and chemokines, without a strong inflammatory factor like lipopolysaccharide, is not precisely understood.

NKT cells are a small population of immune cells in blood (0.2–0.5% in mouse and 0.01—0.5% in human (Berzins et al., 2011), however they can serve as strong inducers of inflammation. NKT cells can rapidly secrete a large amount of cytokines, including Th1, Th2, and Th17 cytokines (Berzins et al., 2011), and these cytokines activate other immune cells (Matsuda et al., 2008). Unlike classic T cells, NKT cells recognize glycolipid antigens presented by CD1d molecules (Kawano et al., 1997). There are two types of NKT cells. Type I NKT cells express a semi-invariant TCR α chain Vα24Jα18 (human) or Vα14Jα18 (mouse) and can be activated with alpha-galactosylceramide (αGC). Type II NKT cells express various TCR α chains and are restricted to CD1d, but cannot be activated with αGC. Mice lacking CD1 (CD1KO) are both type I and II NKT cell deficient. The involvement of CD1d-restricted NKT cells in STEC-mediated disease has never been described. We compared WT and CD1KO mice disease progression following Stx2 injection and observed that CD1KO mice had delayed Stx2-induced pathology.

Historically, it was controversial whether murine glomerular cells such as podocytes and endothelial cells interact with Stx2 for both reports that showed interaction (Morigi et al., 2006) and no interaction (Psotka et al., 2009) existed. On the other hand, human glomerular endothelial cells and podocytes are known to express Gb_3_ and are sensitive to Shiga toxins (Psotka et al., 2009). This has been a problem with the murine model in regards to the similarity to human pathology. We tested direct interaction of Stx2 with murine glomerular cell surface Gb_3_ *in vitro* using a sensitive imaging system, STochastic Optical Reconstruction Microscopy (STORM)—Total Internal Reflection Fluorescence microscope (TIRF) that has ability to image single molecules (Smyth and Shaw, 2008; Dempsey et al., 2011; Liesche et al., 2013). Also, using cellular assays, we tested the ability of Stx2-pre-treated glomerular cells to activate NKT cells *in vitro*. Here for the first time we demonstrate that Stx2 induces CD1d-mediated activation of NKT cells via glomerular cells.

## Materials and methods

### Animals

**Figure 1:**
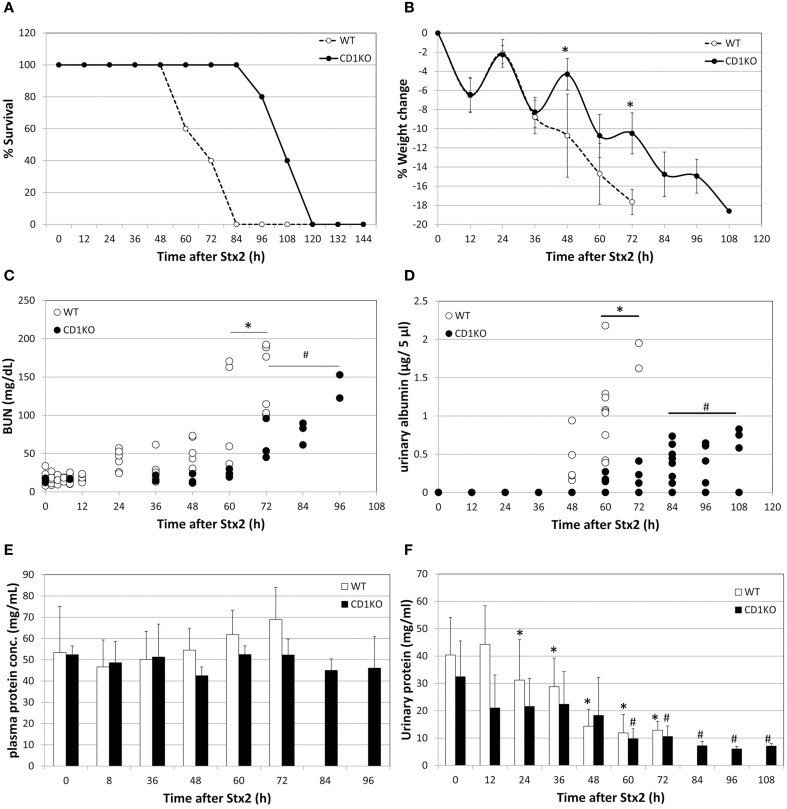
**Delay in Stx2-induced pathology in NKT cell deficient mice. (A)** Percent survival of WT (*n* = 5) and CD1KO (*n* = 5) after Stx2 injection is plotted. Log-rank (Mantel-Cox) test, *p* = 0.0042. **(B)** Percent weight change of WT (*n* = 5) and CD1KO (*n* = 5) after Stx2 injection is plotted. Error bars are standard deviation. Two-Way ANOVA followed by Bonferroni test, ^*^p < 0.05. **(C)** BUN value of each mouse are plotted from WT (*n* = 5 per time point) and CD1KO (*n* = 3 per time point). One-Way ANOVA followed by Tukey test, ^*^p < 0.05 WT compared to 0 h, ^#^*p* < 0.05 CD1KO compared to 0 h. **(D)** Urinary albumin of WT (*n* = 2–9 per time point) and CD1KO (*n* = 4–8 per time point) are plotted for each urine sample obtained at indicated time points. One-Way ANOVA followed by Tukey test, ^*^p < 0.05 WT compared to 0 h, ^#^*p* < 0.05 CD1KO compared to 0 h. **(E)** Averages of plasma concentration of WT (*n* = 5 per time point) and CD1KO (*n* = 3 per time point) from matching samples to **(C)** are shown. Error bars are standard deviation. One-Way ANOVA followed by Tukey test and no significance was detected. **(F)** Averages of urinary protein concentration of WT (*n* = 2 to9 per time point) and CD1KO (*n* = 4 to 8 per time point) from matching samples to **(D)** are shown. One-Way ANOVA followed by Tukey test, ^*^p < 0.05 WT compared to 0 h, ^#^*p* < 0.05 CD1KO compared to 0 h.

Male C57BL/6 (wild type, WT) mice weighing 22 to 24 g were purchased from Charles River Laboratories (Wilmington, MA, USA). Male CD1.1^−/−^ (CD1KO) mice of the matched age were maintained in Webb lab (Carnaud et al., 1999; Park et al., 1999; Hua et al., 2011). Mice were housed in a 12 h/12 h light and dark cycle and given access to food and water *ad libitum*. Mice were injected intraperitoneally 250 ng/kg Stx2 (two times the 50% lethal dose). At this dose of Stx2, 50% of WT mice die at approximately 72 h, and 100% die by 96 h (Figure 1). This dose was chosen based on previous studies with our mouse model of HUS (Keepers et al., 2006). Weight and survival were monitored every 12 h using five mice per strain. In the different set of experiment, at selected time points after injection, three mice per time point were euthanized by CO_2_ inhalation and kidneys were removed. Kidneys collected at 0 h (prior to Stx2 injection) were used for controls. Kidneys were processed for enzyme-linked immunosorbent assay (ELISA) as described below. Mouse blood was withdrawn into a 0.5 M of sodium ethylenediaminetetraacetate (Na_2_EDTA) wetted needle and syringe from their heart at each time point to isolate plasma for blood urea nitrogen (BUN) assay. Blood from 0 h (prior to Stx2 injection) animals were used as controls. All animal procedures were performed in accordance with University of Maryland School of Medicine Animal Care and Use Committee policies (animal use protocol number 0811012).

### Cell culture

Vero cells were purchased from American Type Culture Collection (ATCC, Manassas, VA) and maintained in RPMI1640 media (Life Technologies, Grand Island, NY) supplemented with 10% fetal bovine serum (HyClone/ThermoFisher Scientific, Waltham, MA), 2 mM L-glutamine (Cellgro/Mediatech, Manassas, VA) and 100 U/ml penicillin and 100 μg/ml streptomycin (Life Technologies). Conditionally immortalized murine podocyte and murine glomerular endothelial cells were described previously (Mundel et al., 1997; Akis and Madaio, 2004; Psotka et al., 2009) and cultured in RPMI1640 supplemented with 10% FBS, 2 mM L-glutamine, 100 U/ml penicillin, 100 μg/ml streptomycin and 80 U/ml Interferon-gamma (Sigma-Aldrich, St. Louis, MO) at 33°C as permissive/undifferentiated condition. The cultures were transferred to 37°C and incubated in the media above without IFN-γ and allowed to differentiate for 14 days. Differentiated murine podocytes and glomerular endothelial cells were used in the experiments. Mouse NKT cell lines Vα14^+^DN32.D3 and N38-3C3, and CD1d-specific N37-1A12 (Vα5^+^) were previously described (Lantz and Bendelac, 1994; Brutkiewicz et al., 1995; Burdin et al., 1998; Roberts et al., 2002) and were cultured in Iscove's modified Dulbecco's medium (IMDM) (Life Technologies) supplemented with 5% FBS and 2 mM L-glutamine. Murine L cells transfected with wild-type Cd1d1 cDNA (L-CD1d) or with control vector (L-vector) were described (Chen et al., 1997; Webb et al., 2008) and cultured in Dulbecco's Modified Eagle Medium (DMEM) (Life Technologies) supplemented with 2 mM L-glutamine (Lonza BioWhittaker, Walkersville, MD), 10% FBS (HyClone) and ciprofloxacin (EMD Millipore, Billerica, MA).

### Purification of Stx2 with LPS removal

Purification of Stx2 was kindly provided by Dr. Anne Kane (The Phoenix Lab at Tufts Medical Center, Boston, MA) (Donohue-Rolfe et al., 1989; Stone et al., 2012). LPS was removed from purified Stx2 by Detoxi-gel from Thermo Fisher Scientific (Rockford, IL, USA) by the manufacturer's protocol. A phosphate buffer saline (PBS)-eluted and 0.2 μm filter sterilized Stx2 fraction was tested by Limulus amebocyte lysate assay with detection limit >0.03 endotoxin unit (EU)/ml (Pyrotell, Associates of Cape Cod Incorporated, East Falmouth, MA, USA). Stx2 solutions for culture experiment and mouse injection were determined to contain less than 0.03EU/ml of endotoxin.

### BUN measurement for renal failure evaluation

EDTA-blood was centrifuged at 3000 g for 15 min at 4°C. Plasma was separated from buffy coat and used for BUN assay. BUN assay was performed following directions of the Urea Assay kit (Abnova Taiwan Corporation, Taipei City, Taiwan) with slight modification; plasma was diluted in molecular grade water and samples were incubated for 40 min at room temperature, and incubated with agitation for 10 min at room temperature. Absorption at 520 nm was read using a microplate reader (Synergy HT, Biotek, Winooski, Vermont). Concentration of Urea (mg/dL) was calculated based on a Urea standard curve. Urea concentrations were converted to BUN as stated in the manual: BUN (mg/dL) = [Urea (mg/dL)]/2.14.

### ELISA

Cytokine levels in kidney cell lysates from WT or CD1KO mice treated with Stx2 for the indicated time periods were determined by ELISA. A half kidney was homogenized with 1 ml RIPA buffer consisting 50 mM Tris, 150 mM NaCl, 1% igepal CA-630, 0.5% deoxycholic acid supplemented with protease inhibitor cocktail and 1 mM phenylmethylsulfonyl fluoride (PMSF) (all from Sigma-Aldrich). After centrifugation, resulted supernatant (lysate) was used for the assay. Mouse IL-2 ELISA kit from BD Biosciences (San Jose, CA) or mouse IFN-γ, IL-4 and IL-17 ELISA Max™ kits from BioLegend (San Diego, CA) were purchased and used as per the manufacturer's directions. Kidney lysates were loaded onto ELISA plates in triplicate and cytokine concentrations were determined using a standard curve. The obtained concentrations were normalized to total protein in the sample and represented as pg of cytokine per mg total protein.

### Urine analyses

Urine was collected from mice every 12 h starting at 0 h (prior to Stx2 injection) or the time of tissue harvesting. In the case of dissection, mice were euthanized by CO_2_ inhalation and urine was collected directly from the bladder using a needle and syringe. An equal volume of urine (5 μl) per lane was analyzed by SDS-PAGE with coomassie brilliant blue (CBB) stain along with known amount of bovine serum albumin (BSA). After densitometry of 66 kDa band (albumin) (Sekine et al., 2012), the amount (μg) per 5 μl urine was calculated from BSA standard curve. The protein concentration of urine was analyzed by BCA assay (Thermo Fisher Scientific Inc. Rockford, IL).

### Immunofluorescence stain of Stx2-treated cells

Vero cells, differentiated podocytes and differentiated endothelial cells were trypsinized and seeded on glass bottom petri dishes (MatTek, Ashland, MA). After the attachment, cells were incubated with 20 nM Stx2 for 5 and 15 min at 37°C. No toxin added cells (0 min) were used as toxin negative controls. After the incubation, dishes were placed on the ice, and washed with ice cold phosphate-buffered saline (PBS, Cellgro). Cells were fixed with 4% parafolmaldehyde (PFA, Sigma-Aldrich)/PBS, washed and blocked with 3% Bovine serum albumin (BSA, Sigma-Aldrich)/PBS. In order to detect cell surface localization, cells were not permeabilized. Anti-Gb_3_ rat monoclonal antibody (clone 38–13, Immunotec/Beckman Coulter, Brea, CA) diluted in 1% BSA/PBS incubation was followed by PBS wash and anti-rat IgM-AlexaFluor488 (Life Technologies). After wash, anti-Stx2A mouse monoclonal antibody (11E10, ATCC) diluted in 1% BSA/PBS was followed by PBS wash and anti-mouse IgG-AlexaFluor647 (Life Technologies). Nuclei were stained with 4',6-diamidino-2-phenylindole (DAPI, Life Technologies). Identical dishes were made one for confocal microscopy observation and another for 3D STORM-TIRF observation. Cells were kept in PBS at 4°C until microscopic observation.

### Confocal microscopy

Confocal microscope LSM510 (Carl Zeiss, Thornwood, NY) with Argon (488 nm, 25 mW), Helium Neon (633 nm, 6 mW) and MaiTai (710 nm, 1.5 W) lasers was used to visualize AlexaFluor-488, -647, DAPI and differential interference contrast microscopy (DIC). LSM 5 Image Browser was used to acquire and analyze images.

### Three dimensional STORM-TIRF imaging and analysis

Buffer of the cells was changed to STORM-imaging buffer consisting 50 mM Tris-HCl (pH 8.0), 10 mM NaCl, 10% glucose, 5.6 mg/ml glucose oxidase, 0.17 mg/ml catalase, 100 mM MEA (cysteamine) (all from Sigma-Aldrich). A 256 × 256 pixel field that includes approximately one cell per field was imaged at a time. TIRF degree of 2660 was used in perfect focus 3D STORM setting upon sample bleaching for the certain angles below and above indicated TIRF angle. The sample bleaching is in order to get rid of unspecific signals. Argon (488 nm) and Helium Neon (647 nm) lasers were used in 100% power at acquisition in order to activate all possible fluorescence molecules. A total of 10,000 signals were collected per image. In Nikon NIS-Elements AR software was used to acquire and analyze collected STORM-TIRF images. Diameter of one fluorescence molecule was 0.01 μm. Twelve 488/647 double positive clusters were measured for the size in molecules. The 488 cluster size was calculated as 39.25 ± 14.97 fluorescence molecules per cluster and the 647 cluster size was calculated as 42.83 ± 17.93 fluorescence molecules per cluster. An entire cell was sectioned in 50 nm z-steps (z-slices) from extracellular to intracellular in the range between -500 and 500 nm, and 488/647 double positive objects that fulfill intensity and area size of the positive clusters in each z-slices were accordingly counted.

### *In vitro* NKT activation assay

Differentiated murine podocytes and endothelial cells were treated with or without 1 nM Stx2 for 1 h at 37°C. Cells (5 × 10^5^ per well) were washed extensively (three times) and loaded with 100 ng/ml of α-GalCer purchased from Avanti Polar Lipids (Alabaster, AL) for 2 h at 37°C in IMDM supplemented with 5% FBS and 2 mM L-glutamine from Life Technologies (Carlsbad, CA). After this lipid antigen loading step, cells were washed again and co-cultured with DN32.D3 (type I), N37-1A12 (type II) or N38-3C3 (type I) NKT cell hybridomas (5 × 10^4^ per well) in the same medium. Medium alone and L-vector, a mouse fibroblast cell line transfected with empty vector were used as negative controls. L-CD1d, a mouse fibroblast cell line transfected with CD1d and known to present an unknown, endogenous, strongly activating antigen to NKT cells was used as a positive control. The coculture was set up in triplicate in 96-well microtiter plates for 20 h at 37°C. Following the coculture, supernatants were harvested and IL-2 levels, which are indicative of NKT cell activation were measured by ELISA, as described above (Webb et al., 2006).

### Flow cytometry

Cells were stained in staining buffer (PBS containing 0.5% BSA and 2 mM EDTA) for 30 min at 4°C with phycoerythrin (PE)-conjugated anti-CD1d antibody (clone 1B1) or the respective isotype control from BD Biosciences in accordance with the manufacturer's directions. After staining, cells were washed three times with staining buffer and analyzed immediately by flow cytometry. Data were collected on an LSR II from BD Biosciences and analyzed using FCS Express Version 3 from De Novo Software (Los Angeles, CA).

### Statistical analyses

One-Way ANOVA followed by Tukey test was performed with the time course series samples compared to 0 h value. *P*-values less than 0.05 was determined as significant. Two-Way ANOVA followed by Bonferroni test was used for compare two strains (WT vs. CD1KO), and *p*-values less than 0.05 was determined as significant. All statistical analyses were performed using GraphPad Prism 5.04 software (GraphPad Software, Inc., La Jolla, CA).

## Results

### Stx2-induced pathologies were delayed in CD1KO mice

In order to determine whether NKT cells contribute to Stx2-mediated disease, C57BL/6 (WT) mice and CD1KO (NKT cell deficient) mice were treated with Stx2 (i.p.,) and mortality, weight loss, BUN, urinary albumin, urinary protein, and serum protein were measured (Figure 1). The survival of CD1KO mice injected with Stx2 was prolonged twice as much compared to WT (Figure 1A). Similarly, Stx2-induced weight loss occurred more rapidly in WT mice compared to CD1KO mice, with 10% weight loss occurring in WT mice by 48 h post-treatment, while in CD1KO mice it did not occur until 72 h (Figure 1B). Differences were also detected in renal function. BUN was significantly increased in WT mice 60 h after Stx2 injection, whereas in CD1KO mice, significant increase of BUN was seen after 72 h (Figure 1C). Plasma total protein concentrations in both WT and CD1KO did not change throughout the time course (Figure 1E) emphasizing the significance of BUN increase in Stx2 injected animal. Furthermore, urinary albumin was increased after 60 h of Stx2 injection in WT, whereas it increased after 84 h in CD1KO (Figure 1D). Total urinary protein was reduced in WT 24 h post Stx2 injection, and the reduction was delayed in CD1KO mice, as it occurred after 60 h (Figure 1F). These delays observed in CD1KO strongly suggest NKT cell involvement in the acceleration of Stx2 pathology.

### Stx2-induced renal cytokine response is delayed in CD1KO mice

**Figure 2:**
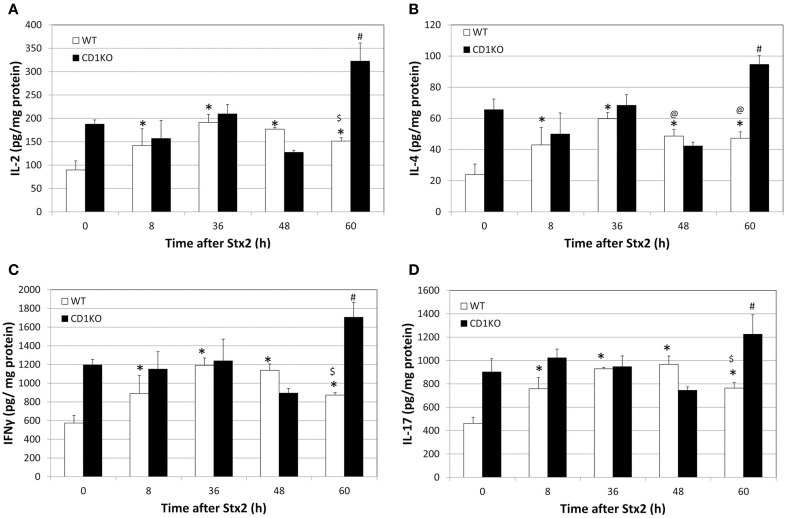
**CD1KO mice show delayed renal cytokine release following treatment with Stx2. (A–D)** WT (*n* = 3 per time point) or CD1KO (*n* = 3 per time point) mice were treated with Stx2 for the indicated time periods. Kidney lysates from both treatment groups at the indicated time points were analyzed by ELISA and **(A)** IL-2, **(B)** IL-4, **(C)** IFN-γ and **(D)** IL-17 concentrations were determined. Results were normalized to total protein and have been represented as pg of cytokine per mg total protein. One-Way ANOVA followed by Tukey test, ^*^p < 0.001 vs. WT 0 h, $*p* < 0.001 vs. WT 36 h, @*p* < 0.05 vs. WT 36 h and ^#^*p* < 0.001 vs. CD1KO 0 h.

Given the delay in Stx2-pathologies in CD1KO mice, we suspected the induction of NKT cell-associated inflammation in kidney. NKT cells produce cytokines such as IL-2, IL-4, IFN-γ, and IL-17. Kidney lysates of Stx2-injected mice were analyzed for these cytokines (Figure 2). In WT mice, IL-2, IL-4, IFN-γ, and IL-17 showed increase after 8 h and peaked at 36–48 h following Stx2 injection. In line with previous markers of Stx2-mediated pathology in CD1KO mice, significant increases in these cytokines were noted later at 60 h. These data suggest that NKT cell activation contributes to accelerate Stx2 renal pathology by promoting inflammation.

### Stx2 binds to mouse renal glomerular cells *in vitro*

**Figure 3:**
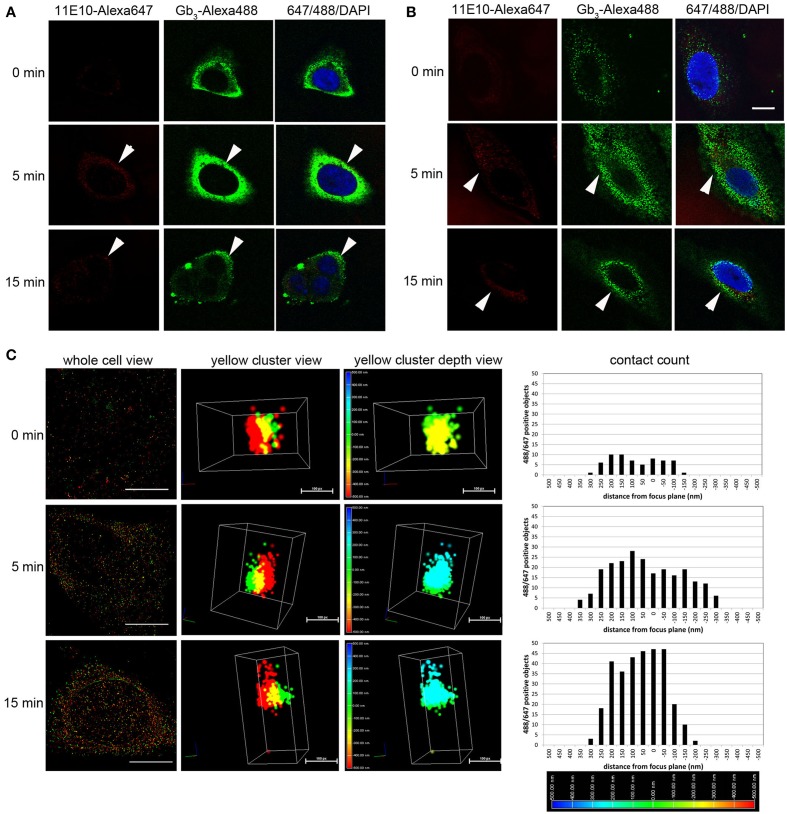
**Stx2 interacts with murine glomerular renal cells *in vitro*. (A)** Murine podocytes or **(B)** Vero cells were incubated with 20 nM Stx2 for 0 (no toxin), 5 and 15 min at 37°C. Stx2 was labeled with monoclonal antibody 11E10 followed by anti-mouse IgG-AlexaFluor 647 (pseudo colored red) and Gb_3_ was labeled with monoclonal antibody 38.13 followed by anti-rat IgM-AlexaFluor 488 (green). Nuclei were stained with DAPI (blue). Samples were observed with confocal microscopy. Representative cells that are chosen from each time point group are shown. Bars indicate 10 μm. Arrowheads point Stx2-AlexaFluor 647 positive cells. **(C)** Murine podocytes were incubated with 20 nM Stx2 for 0 (no toxin), 5 and 15 min at 37°C and labeled as above. Samples were visualized with 3D STORM-TIRF microscopy and analyzed using Nikon Elements software. Whole cell view presents representative cells from each time point. Bars are 10 μm. Yellow spot view presents highly magnified and three dimensionally shown yellow cluster (red = Stx2-Alexa647, green = Gb_3_-Alexa488 and yellow = red and green overlapping pixels). Bars are 100 pixels (px). Yellow cluster depth view depicts the intra or extracellular depth of the yellow cluster. The color-coded depth scale at the left differentiate the distance (nm) from the focus plane (0 nm, green). Blue represents intracellular (up to 500 nm) whereas red represents extracellular (up to -500 nm). Contact count given in the graphs presents amount and depth distribution of green and red contact of a whole cell. The color-coded depth scale at the bottom indicates intracellular (blue) to extracellular (red).

In kidney, the first cell types that NKT cells as well as Stx2 may encounter are glomerular cells, such as endothelial cells and podocytes. We tested the possibility of Stx2 directly interacting with murine glomerular cells by using sensitive fluorescence imaging methods. Murine glomerular podocytes and Vero cells were incubated with Stx2 and the localization of Stx2 (red) and the receptor Gb_3_ (green) was observed by confocal microscopy. Without Stx2, podocytes had none to minimum level of red, whereas Gb_3_ was clearly positive (Figure 3A, 0 min) and the result was similar in Vero cells (Figure 3B, 0 min). In all NKT cell hypbridomas tested, Gb_3_ immunofluorescence was undetectable (data not shown). Stx2 incubation of 5 and 15 min resulted in cell surface Stx2 localization in both podocytes and Vero cells (arrowheads, Figures 3A,B, 5 and 15 min). To observe the precise contact of Stx2 and Gb_3_ molecules at the plasma membrane, 3D STORM-TIRF was utilized. In 3D STORM-TIRF observation, occurrences of Stx2 (red) and Gb_3_ (green) contact can be visualized as yellow clusters (Figure 3C, whole cell view). The occurrence of yellow clusters in Vero cells, podocytes and endothelial cells are shown in Table 1. Because the whole cell view looks at all signals from the top of z-sections and STORM detects individual fluorescence as single molecule, a close up of a yellow cluster shows more precise three dimensional positioning of Stx2 (red) and Gb_3_ (green) (Figure 3C, yellow cluster view). The 360° of a yellow cluster view is presented as a movie (Supplemental Figure 1). In the yellow cluster depth view, the colors are assigned according to the depth of the molecules from outside of the cell as Red (distance -500 nm from plasma membrane) to inside of the cells as blue (distance 500 nm from plasma membrane) (Figure 3C, yellow cluster depth view, 360° appearance in Supplemental Figure 2). For example, 0 min sample in which Stx2 is not added, a yellow cluster that occurs occasionally as background is shown in the depth view that colored as green to yellow (membrane to extracellular). In the samples of 5 and 15 min, representative yellow clusters are blue to green (intracellular to membrane) in the depth view. To show the distribution of yellow clusters along with the depth scale, the yellow clusters from each z-section of an entire cell were counted, and shown in contact count graphs (Figure 3C, contact count). The graph plots depth information in x-axis, and cluster counts in y-axis. The results depicts the distribution of Stx2/Gb_3_ double positive clusters that the longer Stx2 was incubated with the cells, the more cluster counts were obtained and the clusters internalized deeper in the cells. Murine glomerular endothelial cells and Vero cells showed similar results (Supplemental Figure 3). Negative controls such as fluorescence-conjugated secondary antibodies are omitted, had only residual signals of AlexaFluor 647 or 488, and the yellow cluster observation was none to minimum (Supplemental Figure 4).

**Table 1:** **Cells with yellow clusters (488/647 positive clusters) in 3D STORM-TIRF observation^a^**.

	**0 min (%)**	**5 min (%)**	**15 min (%)**
Vero cell	0/3 (0)	2/4 (50)	2/12 (17)
Podocyte	2/3 (66)^b^	1/3 (33)	2/8 (25)
Endothelial cell	2/3 (66)^b^	2/3 (66)	2/3 (66)

a *When more than one yellow cluster is observed in one cell, it is counted as a yellow cluster positive cell. However, the frequency or the total numbers of yellow clusters in the cell or the distribution of the clusters, whether they are intracellular or extracellular, is not reflected in this table. Examples of numbers and distributions of yellow clusters are shown in **Figure 3C***.

b *488/647 overlap clusters are minimum number and/or extracellular*.

### Stx2-treated mouse glomerular cells activate NKT cells

**Figure 4:**
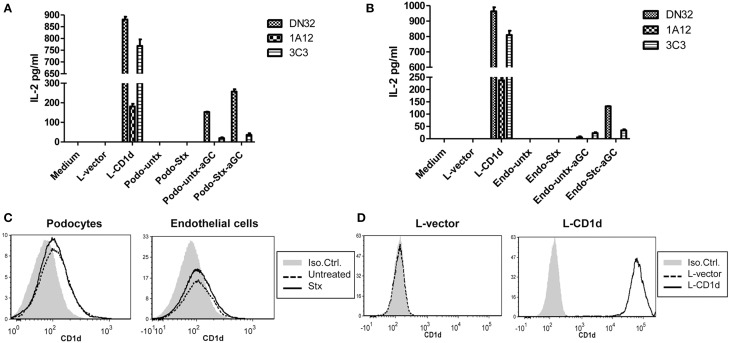
**Stx2-treated murine glomerular renal cells are able to activate NKT cells *in vitro*. (A)** Murine podocytes or **(B)** murine glomerular endothelial cells were treated with (stx) or without (untx) Stx2, washed and incubated either with or without 100 ng/ml of αGC for 2 h. These antigen loaded cells were then washed and cocultured with DN32.D3, N37-1A12 or N38-3C3 NKT cell hybridomas for 20 h. IL-2 was measured in the supernatant by ELISA as readout for NKT cell activation. Medium alone and empty vector-transfected L-cells (L-vector) were used as negative controls, and CD1d-transfected L-cells (L-CD1d) was used as a positive control. **(C)** Murine podocytes and murine glomerular endothelial cells were incubated with (bold line) or without (dashed line) Stx2 and stained with PE-conjugated anti-CD1d antibody and cell surface expression of CD1d was determined by flow cytometry. An isotype control Ab-PE served as a negative control (gray). **(D)** L-vector and L-CD1d were used as negative and positive controls, respectively.

CD1d-restricted NKT cells can be activated by recognizing lipid antigen that is presented in the context of CD1d molecules that are expressed on antigen presenting cells. The ability of Stx2-treated murine renal glomerular cells to induce NKT cell activation was measured by ELISA. Podocytes (Figure 4A) or endothelial cells (Figure 4B) were incubated with or without Stx2, and αGC was added as a lipid antigen, then co-cultured with NKT cells. Stx2-treated cells with αGC induced higher levels of IL-2, compared to the no toxin control in the presence of an exogenous antigen, αGC (*p* < 0.001). Without αGC, both Stx2-treated and non-treated cells did not induce IL-2 production by NKT cells. The positive control, mouse L-cells stably transfected with *mCD1d1* (L-CD1d) induced IL-2 production in all NKT cell hybridomas. In order to determine whether an increase in NKT activation in Stx2-treated cells is due to an increase in CD1d surface expression, flow cytometry was performed with podocytes (Figure 4C) or endothelial cells (Figure 4D). Addition of Stx2 did not change the surface expression of CD1d in podocytes or endothelial cells, suggesting an increase in NKT activation in Stx2-treated renal cells is not due to an increased the levels of CD1d.

## Discussion

We demonstrated for the first time that NKT cells accelerate Stx2-pathology in mice. Mice with normal numbers of NKT cells had higher levels of cytokine early after treatment IL-2, IL-4, IFN-γ, and IL-17 in mouse kidney with subsequent renal failure as well as urine abnormality. In STEC-HUS patients, serum levels of IL-2, IL-4, and IFN-γ were similar to the healthy control group (Shiraishi et al., 2008), however the tissue cytokine levels have not been studied. In the non-human primate that maybe the closest model to humans, IL-2, IL-4, IFN-γ, and IL-17 were not detectable after Shiga toxins injection (Stearns-Kurosawa et al., 2010). In this model, serum IL-12/23 was also not detectable after Shiga toxins injection, but IL-12 mRNA was upregulated in kidneys (Stearns-Kurosawa et al., 2013). This suggests that there is a renal specific inflammation after Shiga toxins injection. NKT cells also produce RANTES and MIP1-α (Chang et al., 2007), which have been implicated in the mouse model of HUS (Keepers et al., 2007; Sauter et al., 2008). NKT cells produce cytokines that can recruit or activate neutrophils (Nakamatsu et al., 2007) and macrophages (Kronenberg and Gapin, 2002), and they are also implicated in Stx2-associated mice renal pathology (Keepers et al., 2007; Roche et al., 2007). Thus, Stx2-associated NKT activation in the kidney may explain rapid renal recruitment of neutrophils and macrophages to induce further inflammation.

*In vitro*, we showed that murine renal glomerular podocytes and endothelial cells express functional CD1d. NKT cells were able to recognize the lipid antigen αGC:CD1d complexes on renal cells and this lead to increased levels of IL-2 when renal cells were treated with Stx2. Activation of NKT cells were only seen in the presence of αGC, this suggests murine renal cells do not present an activating self-antigen (endogenous lipid antigen) after 1 h of Stx2 treatment. However, this may be the result of the short treatment time (1 h) and longer incubations with Stx2 might result in change in the repertoire of lipid antigens. Furthermore, expression levels of CD1d in both types of renal cells were not altered following treatment with Stx2. The mechanism by which Stx2 treatment leads to enhance CD1d-mediated NKT cell activation needs further investigation. In bacterial infection, lipopolysaccharide (LPS) has been shown to interact with toll-like receptor 4 (TLR4) of dendritic cells (DCs) and induce NKT cell activation in the absence of an activating bacterial antigen, but is dependent on the secretion of IL-12 by DC (Brigl et al., 2003; Mattner et al., 2005). As our Stx2 fraction is free of LPS, therefore LPS-induced IL-12 is not responsible for these changes in NKT cell responses; however, our data indicates that Stx2 may induce a co-stimulator(s) factor in renal cells to activate NKT cells.

We presented evidence showing the direct interaction of Stx2 with murine podocyte Gb_3_ in a single molecule imaging analysis known as 3D STORM-TIRF. In traditional light microscopy, the resolution is limited by diffraction and the ability of the microscope to separate objects that are more than 200 nm apart. Whereas, electron microscopy gives higher resolution in nanometer ranges with a tradeoff of longer processing of samples. The STORM technique enables fluorescence imaging to be close to the resolution obtained using electron microscopy by calculating the fluorescence source to its exact position and 3D function allows precise depth information of molecules that are close to plasma membrane in conjunction with TIRF function. To study cell types that are resistant to Shiga toxins but respond by other biological measures, like cytokine production by Stx1-treated human monocytes (Ramegowda and Tesh, 1996), this state of the art imaging technique is an effective tool to provide evidence of the direct interaction of toxin binding to its receptor.

In summary, our data support a model where NKT cells contribute to Stx2-associated renal pathology and lethality. Furthermore, our data suggests that Stx interactions with glomerular cells may promote CD1d-mediated NKT cell activation. Therefore, we hypothesize that NKT cells may accelerate progression to renal failure during STEC infection through Stx2-effects on renal endothelium and podocytes that potentiate NKT-cell activation and result in increased inflammatory signaling. If NKT cells do potentiate inflammation and renal failure during STEC infection, inhibition of NKT-cell activation may be a therapeutic strategy to prevent or delay the progression of STEC-associated systemic disease such as HUS.

### Conflict of interest statement

The authors declare that the research was conducted in the absence of any commercial or financial relationships that could be construed as a potential conflict of interest.
