# Supplementary material for: Natural killer T (NKT) cells accelerate Shiga toxin type 2 (Stx2) pathology in mice
Source: Front Microbiol. 2015 Apr 8;6:262. doi: 10.3389/fmicb.2015.00262 (PMC4389548; doi:10.3389/fmicb.2015.00262)
Supplement: Supplementary file 1 [file DataSheet1.DOCX]

***Supplementary Material***

**Natural killer T (NKT) cells accelerate Shiga toxin type 2 (Stx2) pathology in mice**

**Fumiko Obata ^1,5^*, Priyanka B Subrahmanyam ^1^, Aimee E Vozenilek ^1^, Lauren M Hippler 1, Tynae Jeffers ^1^, Methinee Tongsuk ^1^, Irina Tiper ^1^, Progyaparamita Saha ^1^, Dakshina M Jandhyala ^3^, Glynis L Kolling ^4^, Olga Latinovic ^1,2^, Tonya J Webb ^1^**

^1^Department of Microbiology and Immunology, University of Maryland School of Medicine, Baltimore, MD, USA

^2^Institute of Human Virology, University of Maryland School of Medicine, Baltimore, MD, USA

^3^Department of Molecular Biology and Microbiology, Tufts University, Boston, Massachusetts, USA

^4^Department of Medicine, Division of Infectious Diseases and International Health, University of Virginia, Charlottesville, Virginia, USA

^5^Department of Molecular Pathology, University of Yamanashi Graduate School of Medicine, Chuo, Japan

*** Correspondence:** Fumiko Obata, Department of Molecular Pathology, University of Yamanashi Graduate School of Medicine, Chuo, Japan 1110 Shimokato, Chuo, 409-3898, Japan.

fumikoo@yamanashi.ac.jp

1. **Supplementary Data**

**Supplementary Figure 1. (video 1) A representative close up 300^o^ view of a yellow cluster from Stx2-incubated podocyte (15 min incubation).** Stx2 (depicted in red) and Gb_3_ (green) signals positioned closely with partial overlap (yellow).

**Supplementary Figure S2. (video 2) A depth view of Supplementary Figure 1.** The color-coded depth scale at left indicates green at center (=plasma membrane/focused plane) gradually becomes deep blue at the top (500 nm from the focused plane, toward to intracellular) and red at the bottom (-500 nm from the focused plane, toward to extracellular). Notice the Stx2^+^/Gb_3_^+^ cluster colored between deep blue to green suggesting this cluster is positioned plasma membrane to intracellular.

**Supplementary Figure 3.** **(image 1)** **Stx2 binds to Vero cells and murine glomerular endothelial cells analyzed by 3D STORM-TIRF.** Representative cell analyses of Vero cell without Stx2 (A-C), Vero cell with Stx2 for 5 min (D-F), endothelial cells without Stx2 (G-I) or endothelial cells with Stx2 for 5 min (J-L) are shown. (A, D, G, and J) are whole cell images. Bars indicate 10 μm. (B, E, H and K) are Gb_3_-488/Stx2-647 positive cluster counts in each z-section. X-axis shows the distance from focus plane (=0 nm), in which positive distance indicates intracellular whereas negative distance indicates extracellular. (C, F, I and L) are number of individual AlexaFluor -488 (green) and -647 (red) molecules in each z-section.

**Supplementary Figure 4.**  **(image 2) Negative controls of 3D STORM-TIRF analysis.**  (A) Murine glomerular endothelial cells were incubated with 20 nM Stx2 for 5 min and labeled with anti-Stx2 Ab (11E10) followed by anti-mouse IgG-AlexaFluor 488. AlexaFluor 647 was not added to the sample. (B) Endothelial cells were incubated with 20 nM Stx2 for 15 min and labeled with 11E10 followed by anti-mouse IgG-AlexaFluor 647. AlexaFluor 488 was not added to the sample. Whole cell view, colocalization count and fluorescence molecule count are shown as described in Fig. S3. Bars in the whole cell view indicate 10 μm.
