# Supplementary figures and images for: Natural killer T (NKT) cells accelerate Shiga toxin type 2 (Stx2) pathology in mice
Source: Front Microbiol. 2015 Apr 8;6:262. doi: 10.3389/fmicb.2015.00262 (PMC4389548; doi:10.3389/fmicb.2015.00262)

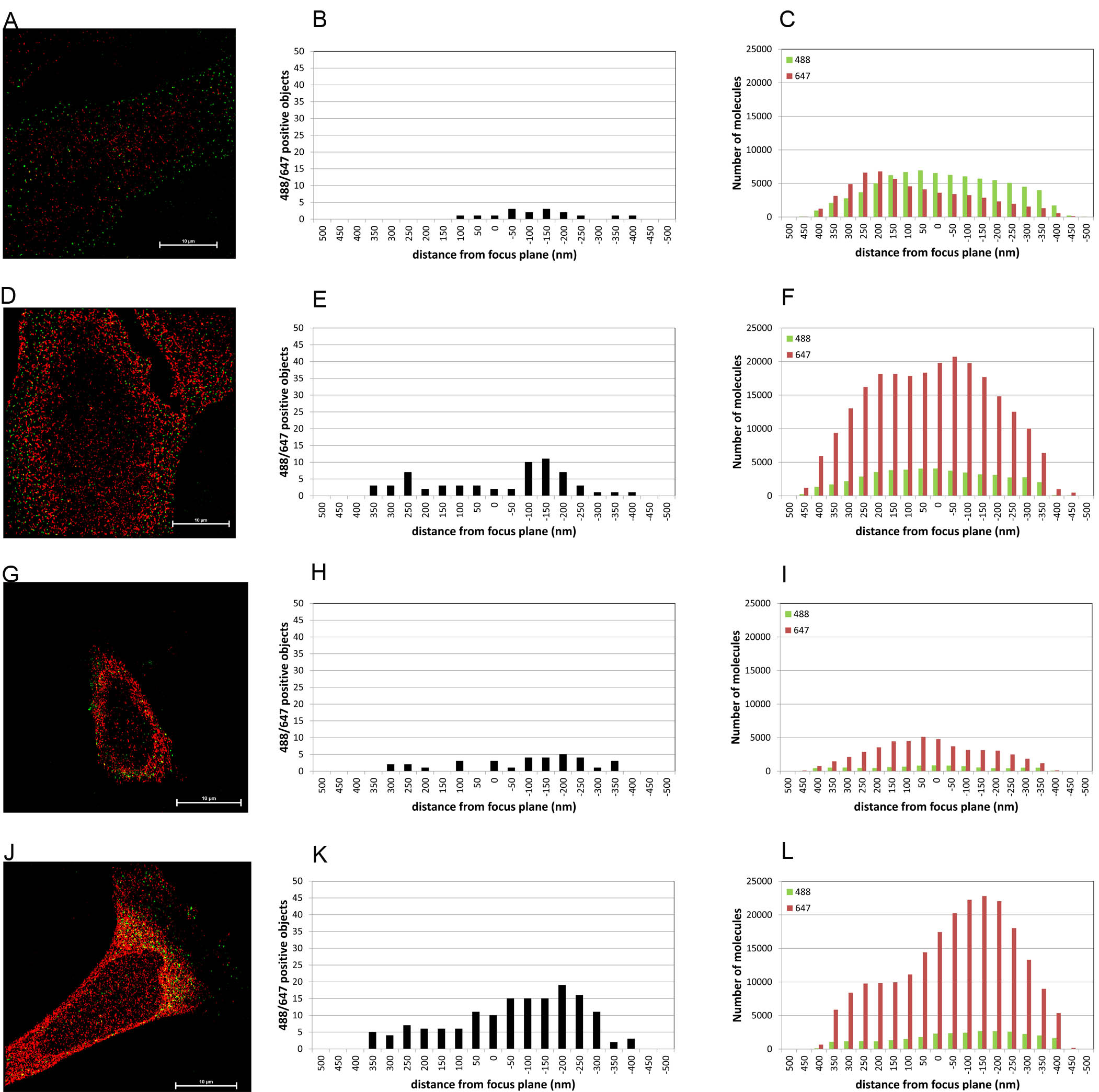

Supplement: Supplementary file 4 [file Image1.JPEG]

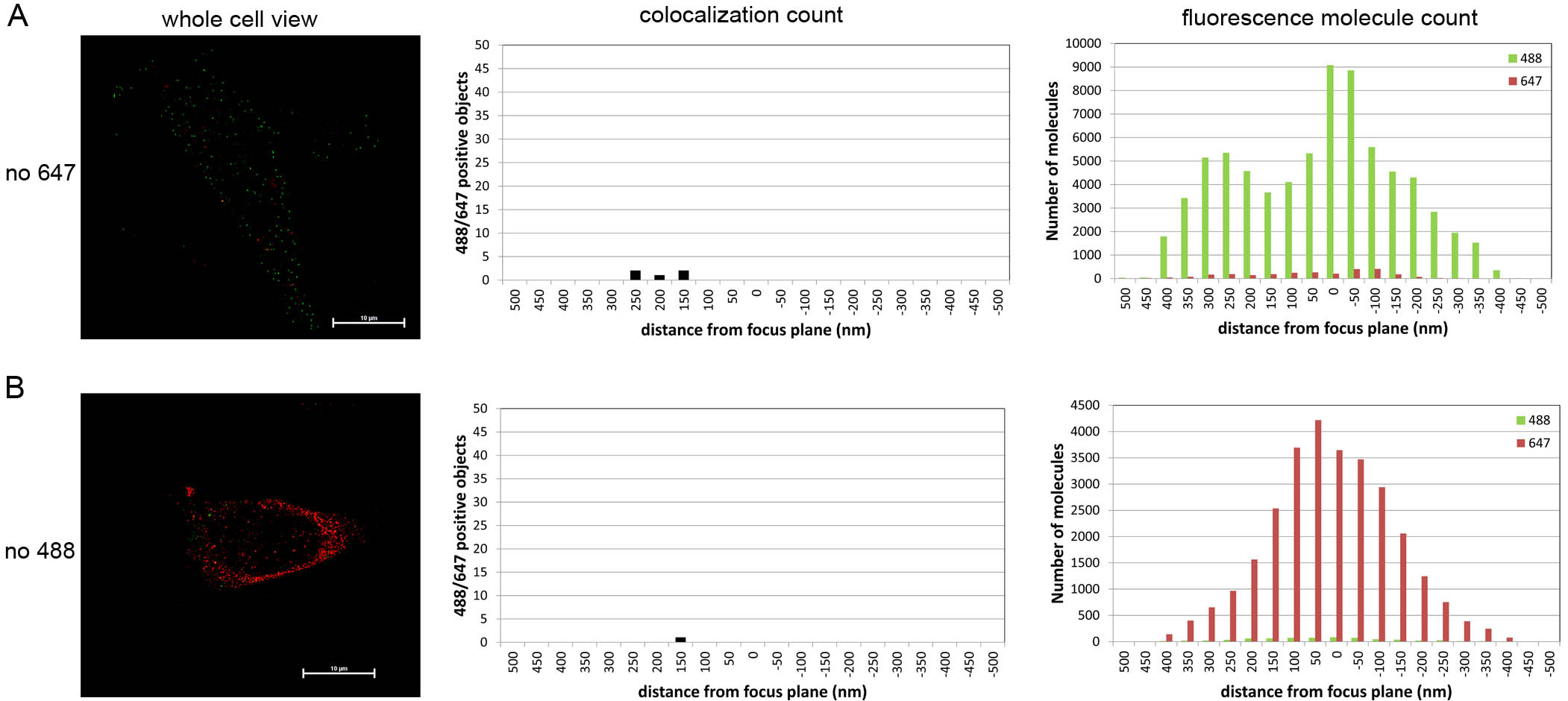

Supplement: Supplementary file 5 [file Image2.JPEG]
